# An early-stage 3D fibroblast-featured tumor model mimics the gene expression of the naïve tumor microenvironment, including genes involved in cancer progression and drug resistance

**DOI:** 10.3389/fonc.2025.1572315

**Published:** 2025-10-27

**Authors:** Francesca Costabile, Subin George, Andrea Facciabene, Gilberto Filaci, Maddalena Mastrogiacomo

**Affiliations:** 1 Department of Internal Medicine and Medical Specialities (DIMI), University of Genova, Genova, Italy; 2 Biotherapy Unit, IRCCS Ospedale Policlinico San Martino, Genova, Italy; 3 Department of Radiation Oncology, Research Division, Perelman School of Medicine, University of Pennsylvania, Philadelphia, PA, United States

**Keywords:** B16F0 melanoma cell, fibroblasts NIH3T3, tumor microenvironment (TME), gene signature, spheroid model

## Abstract

**Introduction:**

The tumor microenvironment (TME) plays a crucial role in cancer progression, yet the interactions between tumor cells and stromal components, such as fibroblasts, remain poorly understood. Traditional two-dimensional (2D) culture models fail to accurately replicate the complexities of the TME, hindering progress in cancer research and drug development.

**Methods:**

This study presents a novel 3D spheroid model, generated using the hanging drop system, that incorporates both tumor cells (B16F10 mouse melanoma) and fibroblasts (NIH/3T3), and aimed at simulating the early-stage TME.

**Results:**

We demonstrate that fibroblasts are essential for ECM deposition, which is absent in spheroids composed only of tumor cells. Co-cultured spheroids exhibited a more organized structure, enhanced ECM deposition (type-VI collagen), and more closely resembled the morphology of native tumors compared to monocultures. RNA sequencing analysis revealed that the gene expression profile of B16F10–NIH/3T3 spheroids closely matched that of in vivo tumors, with 693 genes involved in critical pathways such as “pathways in cancer” and those linked to drug resistance.

**Discussion:**

These findings highlight the importance of fibroblast inclusion in 3D models to replicate the genetic and structural features of the TME. Our spheroid system provides a more accurate representation of early tumor stages and offers a promising platform for drug screening, reducing the need for in vivo models by allowing the selection of the most effective compounds for further testing. This work underscores the potential of 3D culture systems in advancing our understanding of tumor biology and improving the precision of cancer therapeutics.

## 1 Introduction

The tumor microenvironment (TME) is a complex system shaped by direct interactions among different cell types, soluble factors, and extracellular matrix (ECM) (1). Thus, cancer cells form only one component of the TME. The tumor stroma is composed of mesenchymal cells supporting its structure (tumor-associated fibroblasts and macrophages), endothelial cells and pericytes feeding the system, and immune-system cells responding to the cancer insult (T, B, and natural killer cells) (2). Structural cells behave differently in different environments (3). Cancer-associated fibroblasts constitute 5–10% of the total cells of many solid epithelial tumors, such as those of the pancreas, stomach, and breast (4), and they have diverse functions in the TME, including matrix deposition and remodeling, extensive reciprocal signaling with cancer cells, and crosstalk with infiltrating leukocytes (5). They also contribute to carcinogenesis, tumor progression, and metastasis (6, 7).

The ways in which non-cancerous cells and non-cellular components of the TME collaborate with cancer cells and help them to acquire invasive and metastatic features remain unclear. In addition, the specific signals induced during pathological epithelial–mesenchymal transition (EMT) have not been identified (8). Currently, we know that it becomes increasingly important for cancer cells to sustain their growth and functions achieved by recruiting cellular components and modulating their ECM as a tumor develops. Additionally, tumors become increasingly hypoxic with increased size, causing the formation of new vasculature to facilitate the diffusion of nutrients and oxygen to cancer cells through angiogenesis (9, 10). Thus, the TME plays key roles in cancer promotion and maintenance by regulating stemness properties via the activation of key signaling pathways involved in self-renewal, angiogenesis, and the promotion of long-term survival (11). Cancer cells appear to “educate” surrounding (e.g., stromal and immune) cells by secreting signals that recruit, transform, and alter microenvironment functions and activities, in turn facilitating tumor growth and cancer progression (12). Tumors thus leverage ECM remodeling to create a microenvironment that promotes tumorigenesis and metastasis.

Given the emerging importance of the TME in the modulation of cell morphology and function, sophisticated tumor models incorporating TME features are needed to elucidate cellular, molecular, and immunological mechanisms of tumor response and resistance (13, 14). The intensive assessment of *in-vitro* models for the study of tumor complexity has led to the generation of various three-dimensional (3D) culture methods that better mimic *in-vivo* conditions than do usual two-dimensional (2D) methods (15, 16). 3D mono- and co-cultures reproduce *in-vivo* features such as 3D cell morphology, which permits cells to better execute their functions and deposit significantly more ECM (17, 18). These culture techniques also induce cellular phenotype switches from physiological to pathological profiles related to epithelial–mesenchymal transition and cancer-associated fibroblast markers (19–22). Recent studies conducted with 3D cancer models have focused on the cultivation of single cells or spheroidal cell groups, with or without different types of matrix (13–15). The production of cell spheroids is possible when cells have the ability to self-assemble. Hanging drop, scaffold, and hydrogel systems have been developed for this purpose, and spheroid cultures have been applied in drug and nanoparticle testing and disease modeling (23–25). Each approach offers distinct advantages and disadvantages suitable for different research objectives. The hanging drop method relies on gravity-driven self-assembly of cells into spheroids in hanging droplets of cell suspension: surface tension keeps the droplet intact, preventing cells from adhering to a flat surface and promoting cell-to-cell interaction, resulting in the formation of a multicellular aggregate (26). Among the advantages, there surely are its low cost and the easy controlled spheroid size, by adjusting cell number seed. The hanging drop method is moreover very useful for studying cell-cell and cell-extracellular matrix interactions, and this is the reason why we selected it for our study. On the other hand, this system required experience from the operator, to avoid the risk of spheroid loss during media changes. Moreover, the time of observation is limited (2–3 weeks on average) due to the inability of media to penetrate in the core of the spheroid, leading the system to die. Scaffold-based cell culture, instead, provides a structural framework for cells to grow and organize in 3D and can facilitate the delivery of cells, drugs, or growth factors. Scaffolds are typically made of biomaterials (natural or synthetic) mimicking the ECM and allowing cells to attach, proliferate, and migrate within the structured environment. This system supports enhanced cell organization and more realistic cell interactions compared to 2D cultures and hanging drop system. Indeed, scaffolds are extensively useful for tissue engineering and regenerative medicine. The possibility of choosing the more suitable scaffold among many types is definitely an advantage but every scaffold can influence cell behavior differently, requiring careful selection and optimization for each application. Moreover, cells might not well tolerate biomaterials and, because if this matter, most 3D culture system are now build with hydrogels. The hydrogel system utilizes water-swollen, cross-linked polymer networks, as a 3D scaffold, for cell encapsulation or surface coating. Cells can be mixed with hydrogel precursors and encapsulated during gelation, or seeded onto pre-formed hydrogel substrates, allowing them to grow in a 3D environment surrounded by a biomaterial that resembles the ECM (27). Since hydrogels are often composed by decellularized and lyophilized ECM, they are the only system that so far better mimics the native tissue environment, also due to high water content and controllable stiffness. However, some hydrogels may exhibit limited mechanical strength.

Hanging drops, scaffolds, and hydrogels each offer unique capabilities for 3D cell culture. The choice between these methods depends on the specific research question. In this work, we used a hanging drop system to generate spheroids since we are studying the ECM deposition capability and its impact on tumor cells features. Moreover, it is a 3D culture system suitable for the study of the initial stage of the TME and the understanding of biological mechanisms, pathways, cell crosstalk, and morphological changes occurring in different cells present in the tumor bulk. It is scaffold free, which enables the observation of ECM deposition by the cells forming the spheroid. In this study, we also demonstrate the importance of fibroblast inclusion in 3D tumor systems and the similarities of RNA pathways in the system to those in a real model. Compared to previous studies on 3D melanoma spheroids generated by hanging drop system (24, 28), the addition of NIH/3T3 fibroblasts in the proposed model allows a longer lasting experimentation time (up to 3 weeks) and a more realistic tumor cell gene expression, especially for genes involved in therapy resistance. The study findings suggest that a better understanding of tumorigenic ECM remodeling is crucial not only for the discovery of new biological mechanisms, but, more importantly, also for the discovery of new targets and development of new cancer treatments.

## 2 Materials and methods

### 2.1 Cell lines and animals

The B16F10 (ATCC^®^ CRL-6475™, Manassas, Virginia) mouse melanoma cell line was cultivated in RPMI medium supplemented with 10% fetal calf serum and 1% penicillin/streptomycin/fungizone solution. The NIH/3T3 mouse fibroblast line (Interlab Cellular Bank Cell Line Collection, San Martino Hospital, National Institute of Cancer Research, Genova, Italy) was cultivated in complete Dulbecco’s modified Eagle medium with 10% fetal calf serum and 1% penicillin/streptomycin/fungizone solution. One-year-old female C57BL/6 mice (Charles River Laboratories, Wilmington, MA) were used for this study.

### 2.2 Spheroid generation and morphological analysis

B16F10 cells were seeded alone or with NIH/3T3 cells at a ratio of 1:4 (700:3000 cells, due to the difference in proliferation rate) in 20 µL complete Dulbecco’s modified Eagle medium in lidded sterile Petri dishes (29). For the creation of fluorescent spheroids and examination of cell distribution therein, B16F10 and NIH-3T3 cells in co-culture were stained with PKH26 (2 µM/10^6^ cells) and carboxyfluorescein succinimidyl ester (10 µM/10^6^ cells), respectively. The efficacy of staining was evaluated by flow cytometry (LSRFortessa; Becton Dickinson, Franklin Lakes, NJ). Starting from day 5 of culture, the medium was changed as needed.

On day 7, the 3D morphology of the spheroids generated was observed under an FV500 confocal laser scanning microscope, and the classification of Kenny et al. (30) was used to characterize cell organization. Spheroid roundness was measured using the roundness function with ImageJ software (National Institutes of Health, Bethesda, MD, USA; http://rsb.info.nih.gov/ij/).

### 2.3 Animal procedures

The mice were handled according to guidelines conforming to Italy’s current regulations regarding the protection of animals used for scientific purposes. The animal experimentation ethics committee of the National Institute of Cancer Research and the Italian Ministry of Health approved the study procedures (protocol 517: 22418.142). Mycoplasma-free B16F10 cells (*n* = 500,000) in 100 µL phosphate-buffered saline were injected subcutaneously into both flanks of the mice (13). The mice were sacrificed when the tumor volumes [1/2 (length ×width^2^)] reached 1 cm^3^ (31).

### 2.4 Green fluorescent Linterna™ B16F10 cell sorting

The Linterna™ B16F10 cell line (Innoprot, Derio, Spain), with turbo–green fluorescent protein (GFP) at the cytoplasmic level, was used to generate spheroids with red fluorescent NIH/3T3 cells (Innoprot), 2D co-culture with NIH/3T3 cells, and a monolayer. GFP-positive cells were isolated from the cultures using a FACS ARIA IIU-2 sorter. Dead cells were excluded by 7-aminoactinomycin D staining.

### 2.5 RNA isolation and sequencing

Total RNA from 2D cultured cells, spheroids and cells sorted from tumors (n=3) was extracted using Trizol reagent (Invitrogen, Carlsbad, CA, USA) and an RNeasy kit (Qiagen, Hilden, Germany) according to the manufacturer’s instructions. Total RNA concentrations and quality were evaluated for sample inclusion in subsequent *in-vitro* transcription assays based on spectrophotometric absorption ratios of 260/280  > 1.8 (NanoDrop, Wilmington, DE, USA) and RNA integrity numbers > 8.0, determined via electrophoretic analysis (Genewiz, NJ, USA). The RNA was used for next-generation sequencing library generation (Genewiz). Differential expression analysis was performed using NOIseq (32) and GFold (33).

### 2.6 Pathway analysis

A heatmap comparing gene expression in GFP Linterna™ sorted cells from *in-vivo* mouse tumor tissue and 2D and 3D co-cultured cell samples with respective control samples was generated using the heatmap.2 tool of the gplots package (version 3.1.1) in R. The average normalized expression across the three B16F10 control conditions was used to generate the heatmap because the NOISeq R package (version 2.34.0) simulates technical replicates for differential expression analysis when an insufficient number of replicates is available, resulting in slight differences among comparisons. Database for Annotation, Visualization, and Integrated Discovery (a web-based tool for gene annotation and the interpretation of biological meaning) Kyoto Encyclopedia of Genes and Genomes pathway analysis was performed using unregulated genes that overlapped only between the mouse tumor and spheroid samples. To identify these genes, we first selected genes with the same expression levels in the spheroids and *in vivo* model, and then excluded genes with the same expression levels also in the two 2D culture samples. A mean difference (MD) plot (log-intensity ratios vs. log-intensity averages) was generated based on differential expression between GFP B16F10 Linterna™ cells sorted from 3D co-culture with fibroblasts and *in-vivo* tumors generated from the same cell line.

### 2.7 Immunohistochemistry and immunofluorescence analyses

Tissues were embedded in optimal cutting temperature compound, cut into 7-µm-thick sections (unless otherwise specified) with a cryostat (CM3050S; Leica, Wetzlar, Germany), and stained with hematoxylin and eosin (Bio Optica, Milan, Italy) or with Picrosirius Red Stain kit (24901, Polyciences, Warrington, PA), according to the manufacturer’s instructions. For immunofluorescence (IF) analysis, the sections were blocked with 20% goat serum and incubated with the primary antibody type-VI collagen (1:100, ab6588; Abcam, Cambridge, UK) or mTOR (1:500, PA5-34663, Invitrogen, Waltham, MA). The slides were then washed and incubated with Alexa Fluor 594–labeled goat anti-rabbit immunoglobulin G (heavy and light chains, 1:400, A11007; Invitrogen, Waltham, MA] or Alexa Fluor 488–labeled goat anti-rabbit immunoglobulin G (heavy and light chains, 1:800, A11006; Invitrogen). The nuclei were stained with 500 ng/mL 4′,6-diamidino-2-phenylindole (d9542; Sigma-Aldrich). Random fields of each specimen were photographed under a direct microscope, and ECM (type-VI collagen) deposition was assessed on frozen sections obtained on days 7, 14, and 21 of spheroid culture. Quantification of collagen VI signal was assessed by calculating the Integrated Density value via ImageJ software of three different spheroids each group.

### 2.8 Statistical analysis

Graphs were created using the GraphPad Prims software (version 8). Data are reported as means ± standard deviations. Data from pairs of experimental groups were compared using the two-sided Student’s *t* test. *P* values < 0.05 were considered to be significant.

## 3 Results

### 3.1 Fibroblasts are needed for the generation of an early-stage 3D tumor model

**Figure 1:**
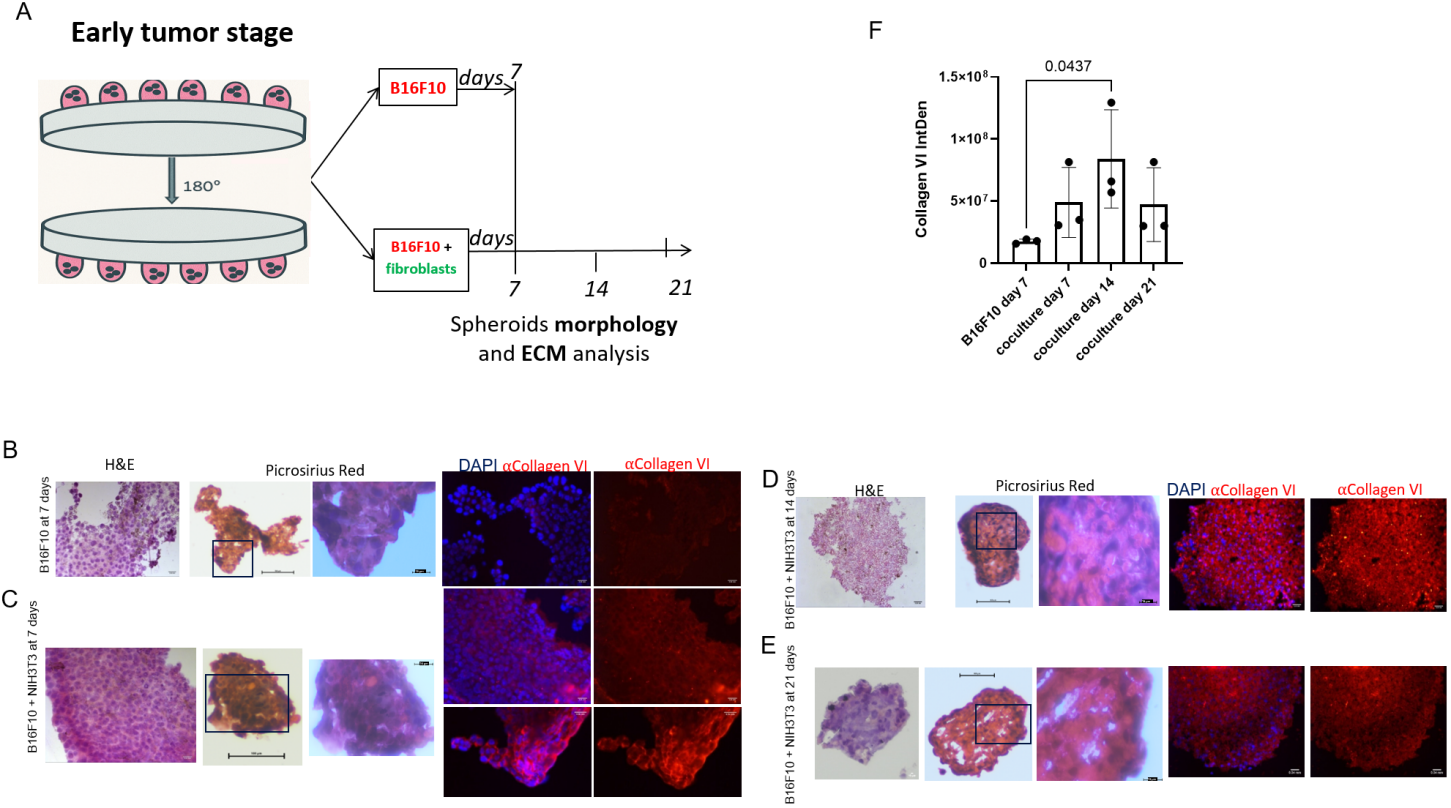
Spheroid generation and ECM deposition. **(A)** Spheroids were generated by B16F10 cells and by B16F10 and NIH/3T3 cells combination. At days 7, 14 and 21 analyses of morphology and extracellular matrix deposition were executed. ECM deposition has been detected by type-VI collagen deposition in B16F10 spheroids at 7 days **(B)** and B16F10–NIH/3T3 spheroids at 7 **(C)**, 14 **(D)**, and 21 **(E)** days, and has been quantified **(F)**. Magnification = 40×. H&E, hematoxylin and eosin. DAPI, 4′,6-diamidino-2-phenylindole. Collagen in spheroids at day 7, 14 and 21 has been analyzed also with Picrosirius Red Staining **(B–E)**.

Using the hanging drop system, B16F10 spheroids, which did not last more than 1 week, and B16F10–NIH/3T3 spheroids were generated to reproduce an early 3D tumor stage model suitable for the study on cell crosstalk and ECM (**Figure 1A**). Structural analysis were assessed every 7 days for 3 weeks. Deposition of ECM was detected through IF analysis and Picrosirius Red Staining. Signals reflecting type-VI collagen deposition were barely detectable in B16F10 spheroids, but clear in co-cultured spheroids (**Figures 1B–E**). Similarly, red staining, representing collagen (**Figures 1B–E**, Picrosirius Red), increases with the addition of fibroblasts to the spheroid model and over time. Quantification of Collagen VI in **Figure 1F** clearly shows the difference of spheroids ECM in presence or not of fibroblasts. Also, nuclear signaling decreased over time, reminding the limitation of the spheroids 3D system. (**Figures 1D, E**).

**Figure 2:**
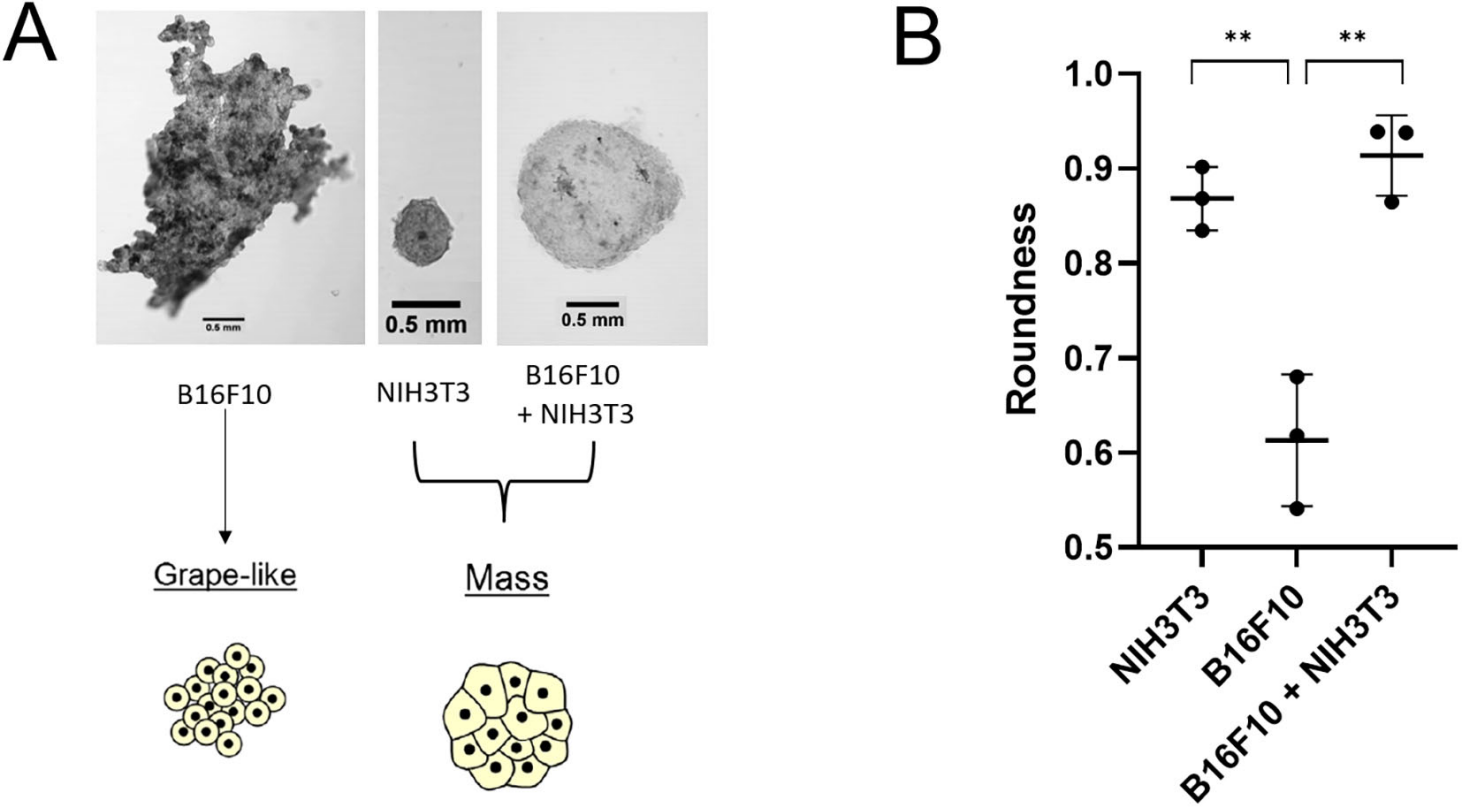
Spheroid morphology. **(A)** Confocal laser scanning microscopic images of representative spheroids made with B16F10 and/or NIH/3T3 cells on day 7. Magnification = 10×. **(B)** Spheroid roundness (mean ± standard deviation, *n* = 9). **p < 0.01 (B16F10 vs. co-culture, *p* = 0.003).

**Figure 3:**
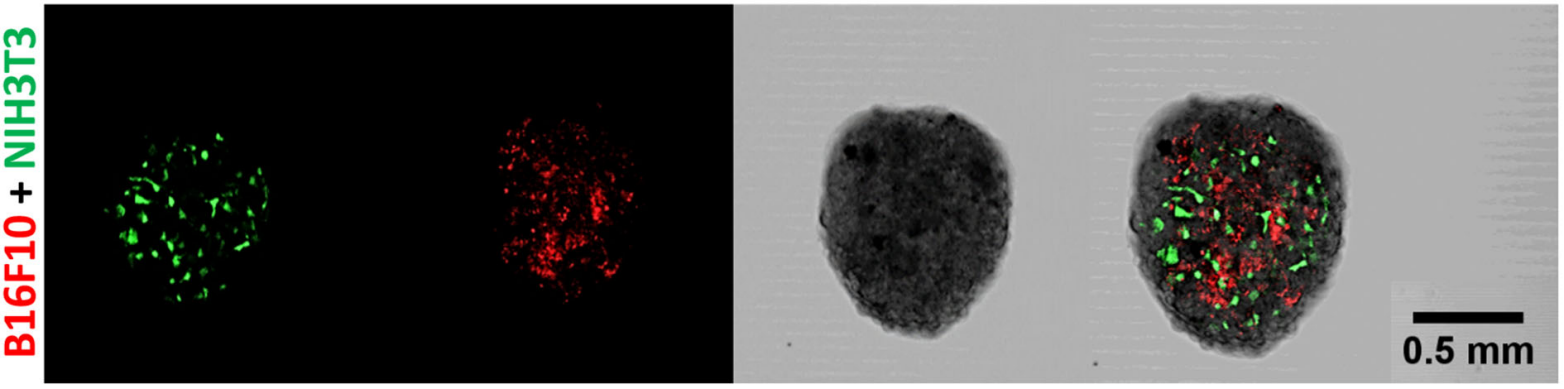
Cell distribution in representative B16F10–NIH/3T3 spheroids (cells stained with PKH26 and carboxyfluorescein succinimidyl ester, respectively) on day 7. Some spheroids were generated with fluorescently stained cells (PKH26 for B16F10 and CSFE for NIH/3T3 cells) for the examination of cell distribution by confocal laser scanning microscopy. Confocal laser scanning microscopic images, magnification = 10×.

As **Figure 2** shows, it is very evident how, beside the deposition of ECM, even the morphology of the single- and two-cell spheroids differed. According to the classification of Kenny et al. (25), each cell line adopts a colony morphology of one of four main classes in 3D culture. These morphologies reflect gene expression profile and protein expression patterns of the cell lines, and distinct morphologies are also associated with tumor cell invasiveness and metastases formation. According to this classification, the B16F10–NIH/3T3 spheroids belong to the “mass class” (roundness index = 0.914), with cells organized regularly around the colony center (**Figure 3**). The B16F10 spheroids belong instead to the “grape-like class” (roundness index = 0.613, *p* = 0.003 vs. B16F10–NIH/3T3 spheroids), with poor cell–cell contact, resulting in a lack of compactness and a grape-like appearance (**Figure 3**). Therefore, the B16F10 spheroids exhibited a lack of robust cell–cell adhesion and the absence of type-VI collagen. Fluorescence examination of B16F10–NIH/3T3 spheroids indicated that the two cell lines were distributed homogeneously in the co-cultured spheroids (**Figure 2**).

### 3.2 The 3D spheroid model shares many biological processes with the *in vivo* TME

**Figure 4:**
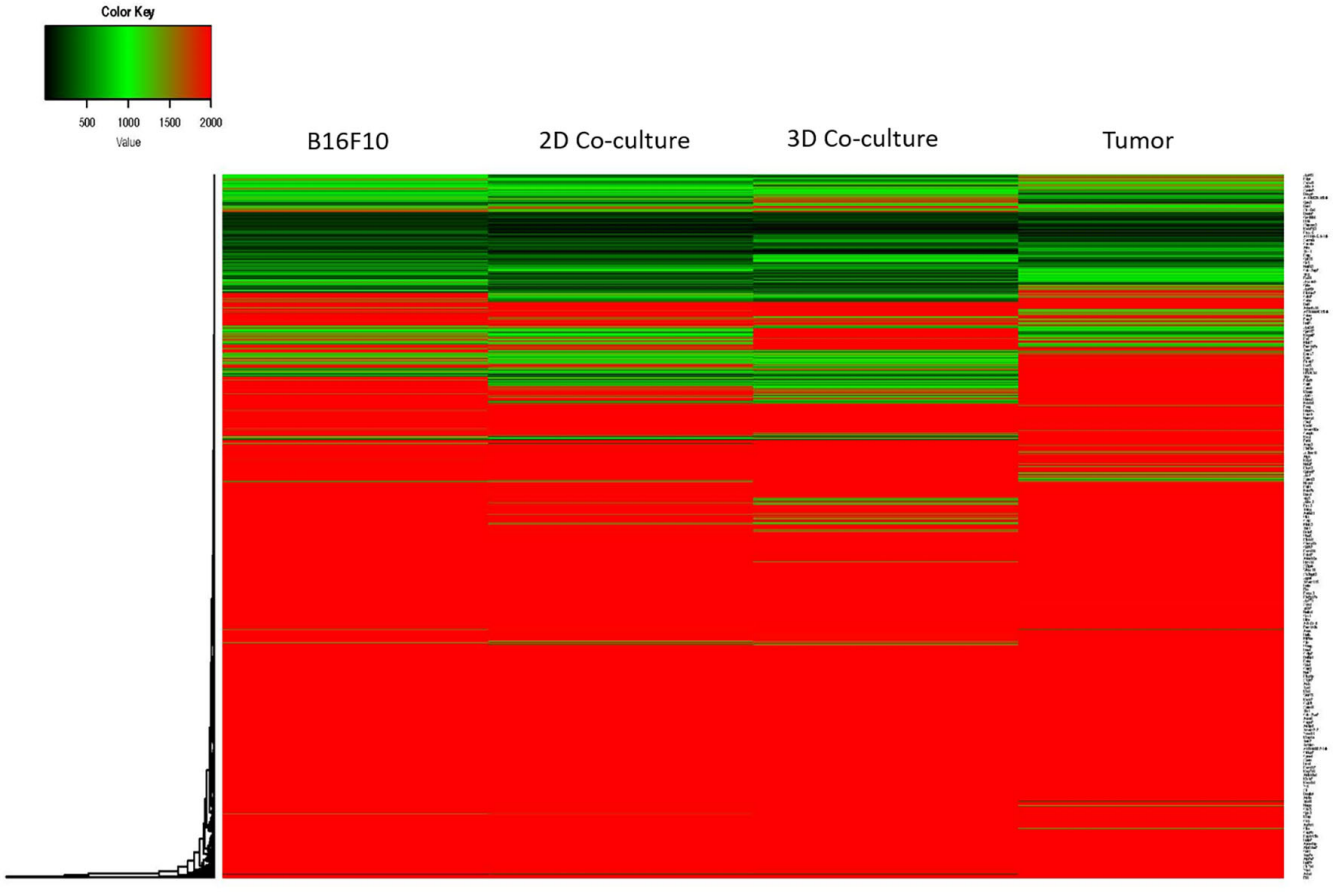
Heatmap of gene expression. The average normalized expression of the B16F10 control conditions from the three comparisons was used to generate the heatmap.

**Figure 5:**
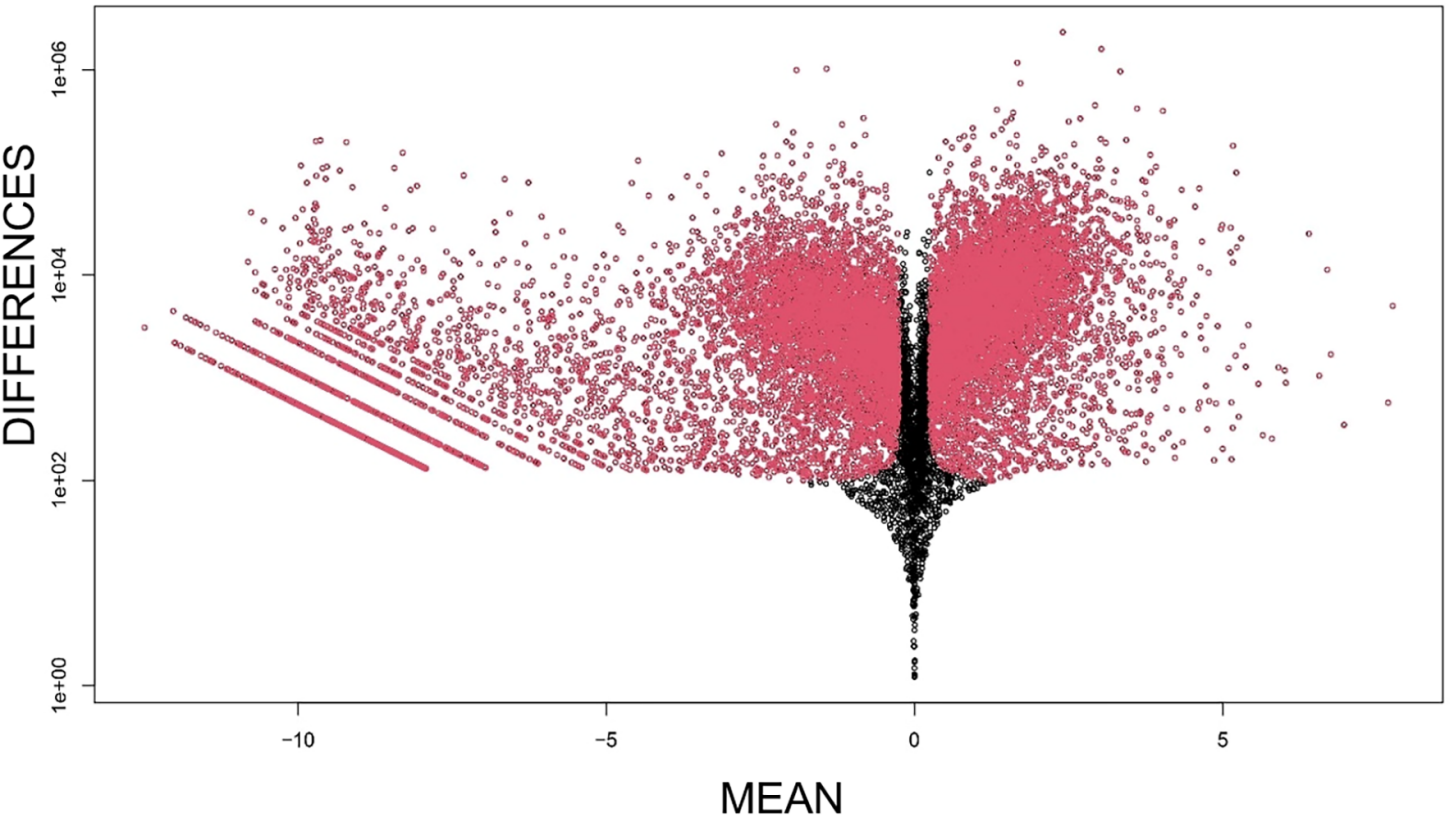
Plot of mean differential gene expression between green fluorescent protein B16F10 LinternaTM cells sorted from three-dimensional co-culture with fibroblasts and in-vivo tumors generated from the same cell line. Black dots, unregulated genes (*n* = 1780); red dots with negative (positive) x-axis values, genes down-regulated (upregulated) in spheroids relative to tumor samples.

**Figure 6:**
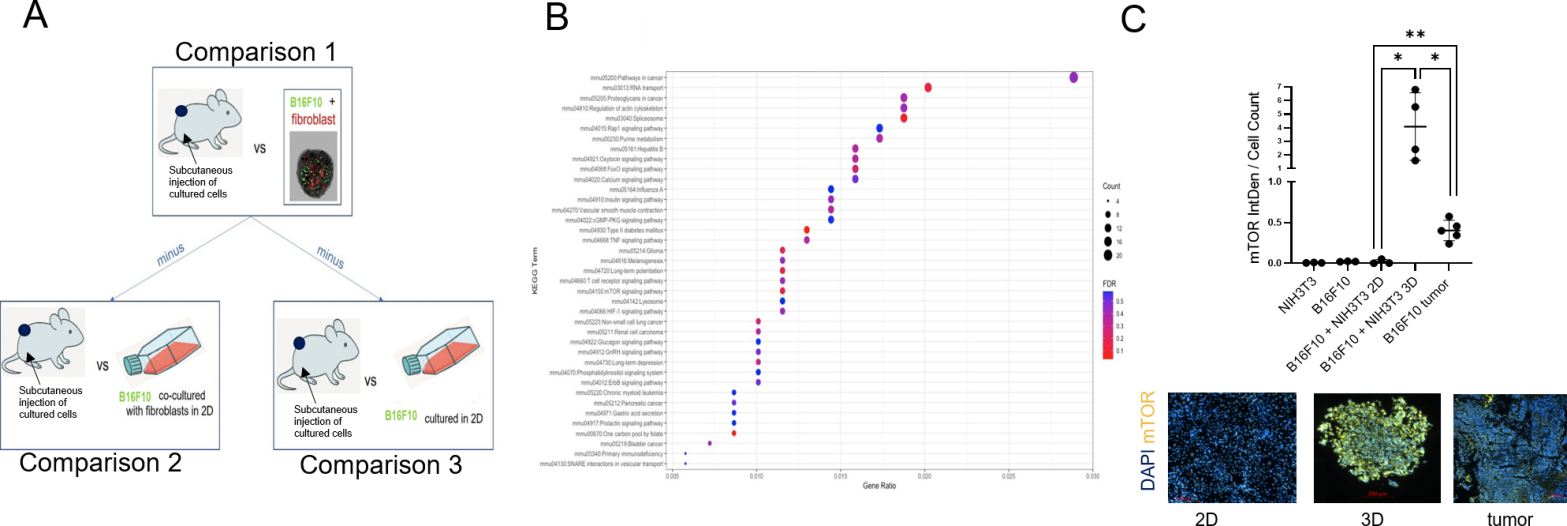
Selection of genes, KEGG pathways analysis and protein validation. **(A)** For each of the 3 comparisons, the genes having the same expression level were selected, generating 3 lists of genes, one for each comparison. All the genes present in the 2 lists of genes generated from comparisons 2 and 3 were removed from the list of genes generated from the comparison 1, obtaining the exclusive gene signature in common between spheroid and *in vivo* model. **(B)** Ratio of the number of genes involved in a pathway to the total of 693 genes. FDR: false discovery rate. **(C)** mTOR protein IF images representing of 3D co-cultures, 2D co-cultures, monocultures and tumor tissue. mTor in yellow and Dapi in blue. Dot plot shows relative mTOR quantification.

To understand the genetic signature beyond the different spheroids’ phenotype, gene expression of B16F10 cultured in the different condition was analyzed and the derived heatmap revealed no evident similarity or difference in gene expression among the four samples compared (**Figure 4**). The MD plot between B16F10 sorted from B16F10–NIH/3T3 spheroids and from *in vivo* tumor showed, instead, that 1780 genes were unregulated, meaning having the same level of expression in the co-culture spheroid model and *in vivo* model (**Figure 5**). In this comparison, 6263 genes were instead downregulated and 5670 genes were upregulated in B16F10 cells cultured in spheroid with fibroblasts compared to *in vivo* tumor. The exclusion from this list of 1780 genes of the genes having the same expression level even in the other two 2D systems (*B16F10* cultured alone and with fibroblasts) allowed us to identify a set of 693 genes with the same expression levels in B16F10–NIH/3T3 spheroids and *in vivo* tumor samples (**Figure 6A**). These 693 genes were involved in 39 Kyoto Encyclopedia of Genes and Genomes pathways, 23.1% of which were associated with the TME. Notably, the “pathways in cancer” term was most enriched (**Figure 6B**, **Table 1**). In particular, the following 20 genes were associated with this term: EGLN1 [the proline hydroxylase mediating degradation of hypoxia-inducible factor α (HIFα) that is associated with tumorigenesis and radioresistance (34)]; RALBP1 [it plays a role in receptor-mediated endocytosis, is a downstream effector of the small GTP-binding protein RAL and mediates multidrug-resistance (35–37)]; FZD3 [the receptor for the wingless type MMTV integration site family of signaling proteins, that is involved in ovarian cancer resistance (38)]; PTGER1 [the prostaglandin E receptor 1 that mediates proliferation of tumor cells (39)]; BRAF [it plays a role in cell growth and division and is tumor drug target (40)]; PRKCA [a protein kinase involved in cisplatin resistance (41)]; ADCY7 [it catalyzes the formation of cyclic AMP from ATP and is abnormally expressed in multiple human cancers (42)]; MTOR [it controls many cell functions, including cell division, survival, and growth, and is involved in cancer drug resistance (43–45)]; CKS1B [it plays a critical role in cell cycle progression, is associated with the pathogenesis of many human cancers and strictly related to drug resistance (46)]; NFKBIA [the NF-kappa-B inhibitor that can provoke drug resistance in cancer if mutated (47)]; RASSF1 [it is a tumor suppressor agent and his inactivation can dysregulate the RAS, Hippo, Wnt and other tumor-related signaling pathways potentially leading to drug resistance (48)]; CDK6 and CDK4 [that regulate cell cycle and are involved in the development of several types of cancer (49)]; GNAQ [part of a trimeric G protein complex is mutated mostly in uveal melanomas (50)]; TRAF5 [his inhibition drives cancer cell apoptosis and improves retinoic acid sensitivity in multiple cancers models (51)]; MAPK1 [the MAPK pathway is responsible for sequential activation of downstream targets, such as MEK and the transcription factor ERK, which control numerous cellular and physiological processes, including organism development, cell cycle control, cell proliferation and differentiation, cell survival, and death: defects in this signaling cascade are associated with cancer development (52)]; EP300 [it regulates cell growth and division, is critical for normal development and has recently been shown as tumor activator, to promote cancer cell proliferation, immune evasion and drug-resistance (53)]; ITGA6 [the transmembrane receptor involved in cell adhesion and signaling which enhances radiation resistance via PI3K/Akt and MEK/Erk signaling (54)]; VHL [his mutation causes cancer and his germline inactivation causes hereditary cancer syndrome (55)]; and RAF1 [pro-oncogene contributing to cell proliferation (56)]. To validate the RNA analysis, we quantified with IF the level of mTOR protein (**Figure 6C**), confirming the presence of the protein exclusively within the actual tumor tissue and the B16F10–NIH/3T3 spheroids.

**Table 1:** KEGG pathways for 693 genes with same expression levels in B16F10 cells sorted from three-dimensional co-culture with fibroblasts and *in-vivo* tumor samples.

Pathway	Genes (*n*)	Pathway	Genes (*n*)
**Pathways in cancer**	20	Melanogenesis7	8
RNA transport	14	T-cell receptor signaling pathway	8
Spliceosome	13	HIF-1 signaling pathway	8
**Proteoglycans in cancer**	13	Lysosome	8
Regulation of actin cytoskeleton	13	**Non-small cell lung cancer**	7
Purine metabolism	12	Long-term depression	7
Rap1 signaling pathway	12	**Renal cell carcinoma**	7
FoxO signaling pathway	11	ErbB signaling pathway	7
Hepatitis B	11	GnRH signaling pathway	7
Oxytocin signaling pathway	11	Phosphatidylinositol signaling system	7
Calcium signaling pathway	11	Glucagon signaling pathway	7
Vascular smooth muscle contraction	10	One carbon pool by folate	6
Insulin signaling pathway	10	**Pancreatic cancer**	6
cGMP-PKG signaling pathway	10	**Chronic myeloid leukemia**	6
Influenza A	10	Gastric acid secretion	6
Type II diabetes mellitus	9	Prolactin signaling pathway	6
**TNF signaling pathway**	9	**Bladder cancer**	5
mTOR signaling pathway	8	SNARE interactions in vesicular transport	4
**Glioma**	8	Primary immunodeficiency	4
Long-term potentiation	8		

Bold text indicates cancer-associated pathways (23.1% of the total).

All these genes play a critical role in cancer progression and more importantly, they are associated to therapy resistance. These results underlay the remarkable differences existing between 2D and 3D tumor culture systems mainly impacting on the *in vitro* re-creation of a TME reliably reminiscent of the *in vivo* original one and the subsequent induction of gene expression patterns related to cancer progression and therapy resistance, crucial aspects in cancer research devoted to identification of pathogenic pathways and target molecules for therapeutic agents.

## 4 Discussion

The study of the TME is becoming essential in the field of oncology. Reproducing tissues *in vitro* should realistically recapitulate the native cell–microenvironment crosstalk, central for the correct functionality. Indeed, the structure and chemical nature of the scaffold material when culturing in 3D play a pivotal role. However, *in vitro* techniques for the generation of models incorporating the ECM are still in a developing stage. Synthetic or semi-synthetic materials have often led to disappointing results due to the difficulty in replicating the sophisticated signals encoded within the native ECM (57). Although formed by the same structural units (i.e., elastin, collagen, hyaluronan, proteoglycans, fibronectin, and laminin), the specific organization and amount of structural units of the ECM vary from organ to organ, making necessary to reproduce a realistic ECM composition to mimic a native environment. In this work, we demonstrated the importance of the natural cell produced ECM for *in vitro* experimentation. We developed a model that mimics the early-stage tumor environment culturing and analyzing spheroids made from tumor cells (B16F10) alone and in co-culture with fibroblasts (NIH/3T3 cells). The system we choose for the generation of the spheroids was the hanging drop system, allowing an undisturbed ECM deposition by fibroblasts. To determinate the abundance of the ECM in our system, we decided to keep track of Collagen type VI, since it is abundant in melanoma and other tumor tissues (58–60). We detected ECM only in the co-cultured spheroids (tumor cells + fibroblasts): indeed only these spheroids lasted for more than 1 week (up to 21 days). Based on these results, we deduce that spontaneous ECM deposition can be obtained only when tumor cells and fibroblasts are both present in a spheroid model, and thus that ECM deposition is fibroblast dependent. Moreover, the B16F10–NIH/3T3 spheroids have a “mass” morphology, whereas the B16F10 spheroids have a “grape-like” morphology due to the lack of type-VI collagen deposition, confirming the fibroblast-dependent nature of ECM deposition. Therefore, fibroblasts are needed for the generation of the early tumor stage 3D model we proposed, because of their major roles as ECM producers and TME organizers (61). Moreover, we observed homogenous distribution of the two cell lines in the spheroids, indicating that they interacted with each other in equilibrium and synergy, without competition, to build an organized structure.

Not only the structural similarity, but we also demonstrated the gene expression likeness between a subcutaneous tumor tissue and a tumor-fibroblast spheroid model. Indeed, a major limitation of *in vitro* 2D monoculture models is the lack of the realistic cancer cell signatures. Cougnoux et al. (62) compared pathway analysis of cancer cells grown in 2D and cells from 3D spheroids: 3D spheroids successfully recapitulated *in vivo* transcriptional states characterized by high expression of genes involved in the ribosome, in the proteosome and in glycolysis/gluconeogenesis. 2D-cultured tumor cells appear to be sensitive to certain drugs that are ultimately not effective in real environments, including in patients with cancer. Moreover, nowadays it is well described the importance of cancer associated fibroblasts in tuning cancer progression and drug resistance (63, 64). Thus, the use of 3D culture models including matrisome cells like fibroblasts is essential to improve our understanding of tumor biology and the precision of drug screening. We confirmed the gene expression similarity between the co-cultured spheroid and the native tumor microenvironment particularly regarding genes involved in cancer-related pathways. We even validated mTOR protein levels and demonstrated that it was detectable exclusively in the 3D system including both cell lines, as well as in the actual tumor tissue, compared to single cell line culture, or 2D co-culture. The mTOR protein level is higher in the proposed 3D model compared to the actual tumor tissue most probably because just cancer cells and fibroblasts compose the 3D system while the actual tumor tissue includes many other cell type and matrix where mTOR is not present. This means that we are highlighting the real features of cancer cells in the proposed system, but the heterogeneity of the whole TME is still not well represented yet.

These results highlight the importance of 3D models including fibroblasts mimicking of the TME for tumor experimentation. The spheroid model we proposed is therefore eligible for a realistic early tumor stage *in vitro* system incorporating tumor cells, tumor associated fibroblast and ECM, that all together tunes the genetic expression towards a more realistic environment, incorporating tumor progression gene expression and drug resistance features. However, the tumor heterogeneity and especially the immune system interaction are missing in the proposed model. A more sophisticated and complete system, perhaps with the incorporation of endothelial cells and functioning vessels, would allow the better media perfusion, for a longer lasting system, and the possibility to incorporate the immune system component, to test immunogenicity and immune cells response to different challenges. The increase utilization of this 3D system will furthermore reduce significantly *in vivo* experimentations, being a model for screening a considerable number of drugs and select the only ones with promising results for the next step of screening *in vivo (*65).

## Data Availability

The data presented in the study are deposited in the GEO repository, accession number GSE294100.
